# Cerebral Augmentation Effect Induced by External Counterpulsation Is Not Related to Impaired Dynamic Cerebral Autoregulation in Ischemic Stroke

**DOI:** 10.3389/fneur.2022.784836

**Published:** 2022-05-03

**Authors:** Li Xiong, Xiangyan Chen, Jia Liu, Lawrence Ka Sing Wong, Thomas W. Leung

**Affiliations:** ^1^Clinical Trials Centre, The Eighth Affiliated Hospital of Sun Yat-sen University, Shenzhen, China; ^2^Department of Medicine & Therapeutics, The Chinese University of Hong Kong, Shatin, Hong Kong SAR, China; ^3^Department of Health Technology and Informatics, The Hong Kong Polytechnic University, Shatin, Hong Kong SAR, China; ^4^Shenzhen Institute of Advanced Technology, Chinese Academy of Sciences, Shenzhen, China

**Keywords:** cerebral blood flow, external counterpulsation, dynamic cerebral autoregulation, transfer function analysis, ischemic stroke

## Abstract

**Background and Purpose:**

Dynamic cerebral autoregulation is impaired after ischemic stroke. External counterpulsation (ECP) augments the cerebral blood flow of patients with ischemic stroke by elevation of blood pressure (BP). We aimed to investigate if cerebral augmentation effects during ECP were associated with impaired dynamic cerebral autoregulation in patients after acute ischemic stroke.

**Methods:**

Forty patients with unilateral ischemic stroke and large artery atherosclerosis in the anterior circulation territory within 7 days from symptom onset and eighteen healthy controls were recruited. We monitored changes in mean flow velocity over both middle cerebral arteries (MCA) by transcranial Doppler (TCD) before, during, and immediately after ECP. Cerebral augmentation index was MCA mean flow velocity increase in percentage during ECP compared with baseline to evaluate the augmentation effects of ECP. Spontaneous arterial BP and cerebral blood flow velocity in both bilateral MCAs were recorded using a servo-controlled plethysmograph and TCD, respectively. Transfer function analysis was used to derive the autoregulatory parameters, including phase difference (PD), and gain.

**Results:**

The cerebral augmentation index in patients with stroke was significantly higher on both the ipsilateral and contralateral sides than that in controls, while the PD in patients with stroke was significantly lower on both sides than those in controls (all *P* < 0.05). The cerebral augmentation index did not correlate with PD and gain on either the ipsilateral or contralateral side of patients with stroke or in controls (all *P* > 0.05). The cerebral augmentation index of patients with stroke was significantly related to mean BP change on the ipsilateral side (*R*^2^ = 0.108, *P* = 0.038).

**Conclusion:**

The degree of ECP-induced cerebral augmentation effects as measured by the cerebral augmentation index did not correlate with the magnitude of impaired dynamic cerebral autoregulation.

## Introduction

In the traditional view, cerebral autoregulation ensures the constancy of cerebral blood flow to the brain as the systemic blood pressure (BP), and hence, cerebral perfusion pressure changes over a wide range. However, considering the compelling evidence currently available, cerebral blood flow regulation is far more pressure-passive in nature than traditionally believed. Indeed, cerebral blood flow will not necessarily remain stable in some physiological/clinical conditions (1). Cerebral autoregulation is impaired after ischemic stroke (2, 3). The brain becomes more vulnerable to ischemic damage caused by changes in systemic BP or intracranial pressure. Although the management of BP following acute ischemic stroke remains controversial, the available clinical data suggest that the use of BP augmentation may improve perfusion to ischemic tissue, which can result in at least short-term neurological improvement (4, 5).

External counterpulsation (ECP) is a noninvasive and well-established method for ischemic heart disease (6–8). ECP operates by applying electrocardiography-triggered diastolic pressure to the lower extremities through air-filled cuffs. The diastolic augmentation of the blood flow and the simultaneously decreasing systolic afterload increases the blood flow of vital organs such as the heart, brain, and kidneys (9, 10). Our pilot study showed that ECP is feasible for patients with ischemic stroke with large artery disease by improving neurological deficits (11). ECP may improve cerebral perfusion and collateral blood supply in ischemic stroke by augmenting BP and cerebral blood flow velocity (CBFV) (12). Without cerebral autoregulation, CBFV would passively follow BP. The cerebral augmentation effects induced by ECP possibly worked *via* impaired cerebral autoregulation. Furthermore, we first proposed the cerebral augmentation index to evaluate the degree of cerebral augmentation effects induced by ECP. Recently, we found that the higher cerebral augmentation index on the side ipsilateral to the infarct was independently correlated with an unfavorable functional outcome after acute ischemic stroke (13).

Without too much cooperation from patients, transfer function analysis (TFA) is a frequently used method to assess dynamic cerebral autoregulation using spontaneous oscillations in BP and CBFV despite the controversies and variations in its interpretation from different research groups (14, 15). TFA quantifies cerebral autoregulation in the parameters phase difference (PD), gain, and coherence with the assumption that cerebral autoregulation is a linear control system. It is based on the concept that cerebral autoregulation minimizes the effect of dynamic BP fluctuations on CBFV, which is reflected by reduced low-frequency gain and phase-lead of CBFV over BP (16, 17). Without cerebral autoregulation, CBFV would passively follow BP, and TFA would show constant gain and zero phases across the low-frequency band. In acute ischemic stroke, recent meta-analyses of TFA parameters, obtained from spontaneous fluctuations of BP at rest, have demonstrated that PD and the autoregulation index can show highly significant differences in comparison with healthy controls, while less clear-cut results were obtained for gain (18).

Therefore, in this study, we aimed to investigate whether the degree of cerebral blood flow augmentation effects induced by ECP was related to the magnitude of impaired dynamic cerebral autoregulation in patients with ischemic stroke, assessed by cerebral augmentation index and PD and gain, respectively.

## Methods

### Subjects

Patients with unilateral ischemic stroke in the anterior circulation territory and large artery occlusive disease with a good acoustic window within 7 days of stroke onset in Prince of Wales Hospital in Hong Kong between November 2011 and December 2013 were recruited. Diagnosis of stroke was made based on the definition of the WHO, and ischemia was confirmed by computerized tomography or MRI. All patients were examined by TCD, duplex ultrasound, or magnetic resonance angiography to verify with intracranial or extracranial large artery stenosis (moderate stenosis or > 50% diameter reduction). Based on our previous study, (19). we excluded patients with evidence of cardioembolic strokes such as atrial fibrillation and rheumatic heart disease, evidence of hemorrhage on brain computerized tomography, evidence of arteriovenous malformation, arteriovenous fistula or aneurysm, a history of intracerebral hemorrhage, brain tumor or malignancy, sustained hypertension (systolic > 180 mmHg or diastolic > 100 mmHg), severe symptomatic peripheral vascular disease, evidence of co-existing systemic diseases such as renal failure (creatinine > 300 μmol/L, if known), cirrhosis, thrombocytopenia [platelet count <1,00,000/mm (3)] and severe dementia of psychosis as well as pregnant women. Patients with tissue plasminogen activator (tPA) and thrombectomy intervention were excluded due to their effects on dynamic cerebral autoregulation (20, 21). Healthy volunteers without a history of cerebrovascular events and risk factors were recruited as control subjects. Each healthy subject underwent TCD and carotid duplex to rule out large artery occlusive disease. Written informed consent was obtained from all participants prior to enrolment. This study was approved by the local medical ethics committee (Joint CUHK-NTEC Clinical Research Ethics Committee).

### ECP TCD Monitoring

All the participants were asked to lie in the supine position for 15 mins prior to the beginning of data acquisition. ECP was performed using the Enhanced External Counterpulsation system, model number MC2 (Vamed Medical Instrument Company, Foshan, China). The treatment pressure of ECP was 150 mm Hg. TCD monitoring was performed using the ST3 TCD system (Spencer Technologies, Seattle, WA). The patients lay on the ECP treatment bed and their legs were wrapped with 3 pairs of air cuffs. Two 2-MHz probes were mounted on a head frame, which was fitted individually and worn on the head of patients. M1 segments of bilateral MCAs were insonated at the depth of highest mean flow velocity between 50 and 60 mm. We recorded bilateral CBFV of bilateral MCAs and continuous beat-to-beat arterial BP using a finger plethysmograph using the Task Force Monitor system (CNSystems Medizintechnik AG, Graz, Austria) before and during ECP, respectively, for 3 mins. The cerebral augmentation index was used to assess the augmentation effect of ECP, which was calculated by the increase in the percentage of mean flow velocity during ECP compared with baseline (12).

### Dynamic Cerebral Autoregulation Measurement and Data Analysis

Continuous CBFV of bilateral MCAs *via* TCD and continuous beat-to-beat arterial BP were recorded simultaneously from each subject in the supine position for 10 mins. The recorded data were then used to evaluate dynamic cerebral autoregulation by the method of TFA (15, 22). The data were post-processed using MATLAB (MathWorks, Inc, Natick, MA). A cross-correlation function between arterial BP and CBFV was used to align the raw data in order to eliminate the possible time lags. The signals were then down-sampled to 1 Hz after the application of an anti-alias filter with a cutoff frequency of 0.5 Hz. The dynamic relationship between arterial BP and CBFV was assessed by TFA with an algorithm used in previous studies (23). PD, gain, and coherence within a low-frequency range, 0.06 to 0.12 Hz, were then derived from TFA to evaluate dynamic cerebral autoregulation. The autoregulatory parameters were accepted for further statistical analysis only when coherence was higher than 0.4 within 0.06 to 0.12 Hz.

### Statistical Analysis

The mean flow velocity of MCA was automatically recorded by the TCD system, which was the mean value of the area under the envelope curve in a cardiac cycle. Data of patients with stroke were analyzed based on the side ipsilateral or contralateral to the infarct. Mean CBFV, cerebral augmentation index, PD, and gain on the right and left sides in the control group were averaged and used. Continuous data were analyzed by independent-sample Student *t*-tests when there was a normal distribution and by the Mann–Whitney test, if there was a skewed distribution between both sides of stroke and control groups. Category data were analyzed by the Pearson χ^2^ test or Fisher's exact test. The paired *t*-test was used to compare the difference in CBFV between baseline and during ECP in each group. Pearson correlation analysis was performed between cerebral augmentation index and PD or gain in the two groups as well as between cerebral augmentation index and BP changes in the stroke group by SPSS V.24.0 (SPSS, Inc, Chicago, IL). Differences with *P* < 0.05 were considered significant.

## Results

Forty ischemic stroke patients with large artery disease and eighteen healthy controls were recruited. There were more men in patients with stroke (36 men [90%]) compared with controls (9 men [50%]) (*P* = 0.002). The mean interval from stroke onset to examination was about 5 days, and the median admission National Institutes of Health Stroke Scale score in the patient group was 5 (Table 1). Among the forty stroke patients with large artery disease, 85% (34 cases) had intracranial large artery disease (MCA involved in 21 cases, intracranial internal carotid artery in 9 cases, and anterior cerebral artery in 4 cases) and two patients had extracranial large artery disease. A total of 4 patients had both intracranial and extracranial large artery disease.

**Table 1:** Baseline characteristics.

**Parameters**	**Stroke patients** **(*n* = 40)**	**Healthy controls (*n* = 18)**	***P***
Gender (male/female)	36/4	9/9	0.002^*^
Age (mean ± SD), years	65.0 ± 10.6	61.1 ± 4.5	0.051
Day from symptom onset to recruitment (mean ± SD, d)	5.2 ± 3.0	NA	
Admission NIHSS (median, range)	5 (4–9)	NA	
Hypertension, *n* (%)	35 (87.5)	NA	
Diabetes, *n* (%)	18 (45.0)	NA	
Ischemic heart disease, *n* (%)	4 (10.0)	NA	
Hyperlipidemia, *n* (%)	20 (50.0)	NA	
Previous stroke, *n* (%)	11 (27.5)	NA	
Smoker, *n* (%)	21 (52.5)	NA	
Drinker, *n* (%)	7 (17.5)	NA	
Intracranial large artery disease, *n* (%)	34 (85.0)	NA	
Extracranial large artery disease, *n* (%)	2 (5.0)	NA	
Intra- and extracranial large artery disease, *n* (%)	4 (10.0)	NA	
Left side infarct, *n* (%)	24 (60.0)	NA	
Right side infarct, *n* (%)	16 (40.0)	NA	

*NIHSS, National Institutes of Health stroke scale*.

* *Pearson χ^2^ test*.

**Figure 1:**
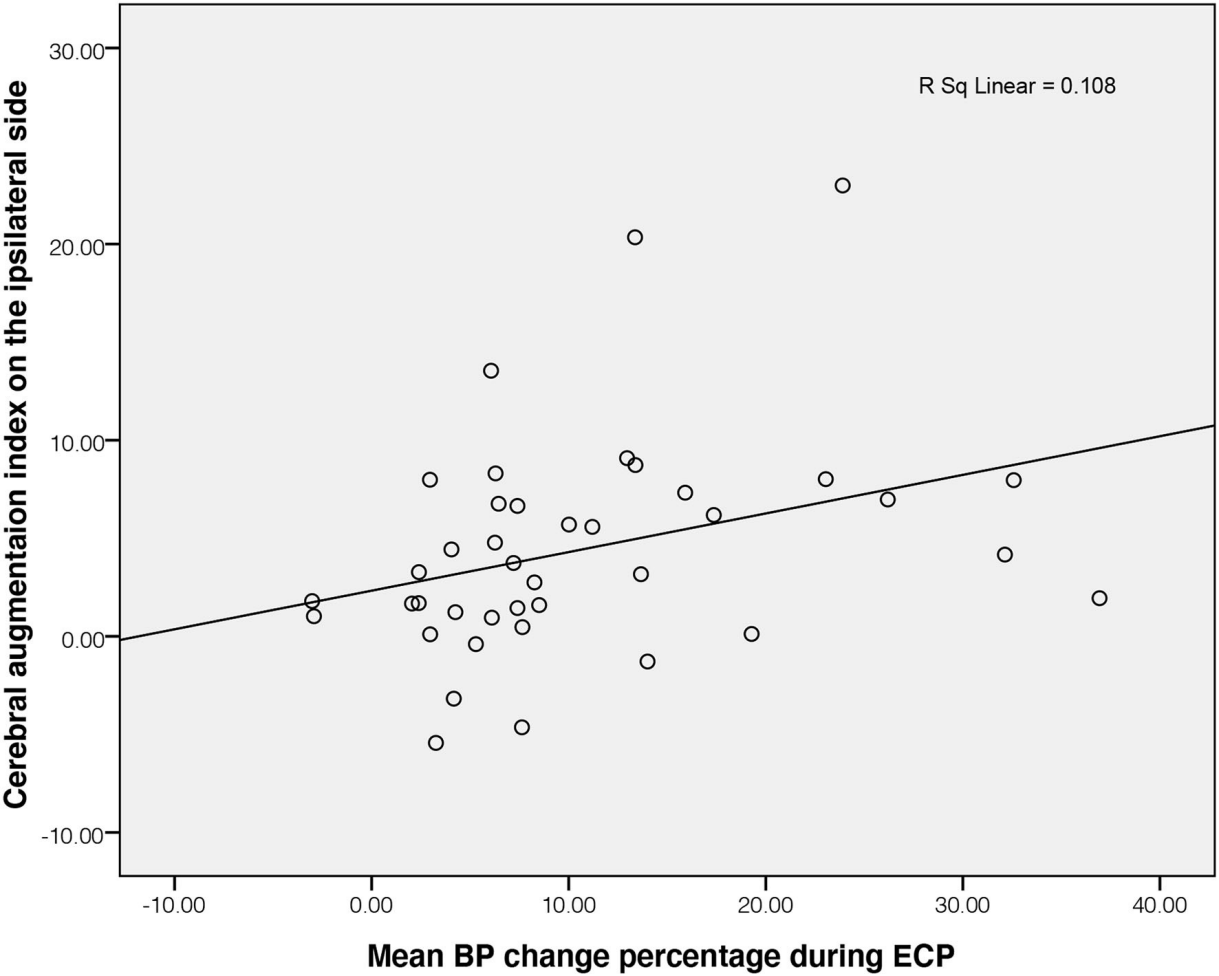
Correlation of BP change and ipsilateral cerebral augmentation index in patients with stroke. BP, blood pressure; ECP, external counterpulsation.

Baseline mean CBFV of patients with stroke on both sides showed no significant difference in comparison with healthy controls (*P* = 0.854 on the ipsilateral side; *P* = 0.162 on the contralateral side). There were no differences in all parameters between the ipsilateral and contralateral sides in the stroke group (all *P* > 0.05). The mean CBFV of patients with stroke significantly increased during ECP as shown in Table 2. In the control group, mean CBFV did not change significantly during ECP from baseline. The cerebral augmentation index of stroke patients was much higher than that of controls. In the patient with stroke group, PD was significantly lower on the ipsilateral side (32.71 ± 39.13 degrees) as well as the contralateral side (28.59 ± 36.87 degrees) than that in the control group (52.64 ± 13.94 degrees, all *P* < 0.05), but there was no significant difference in gain on both sides between the two groups. The cerebral augmentation index did not correlate with PD or gain for either the ipsilateral or contralateral side in the stroke group (all *P* > 0.05). The correlation between cerebral augmentation index and PD was borderline significant in the control group (*P* = 0.05). During ECP, the cerebral augmentation index of patients with stroke was related to the mean BP change on the ipsilateral side (*R*^2^ = 0.108, *P* = 0.038, Figure 1). There was no significant correlation between cerebral augmentation index and BP change in controls (*R*^2^ = 0.349, *P* = 0.131) as well as on the contralateral side in patients with stroke (*R*^2^ = 0.041, *P* = 0.801).

**Table 2:** Cerebral augmentation effects of ECP and dynamic cerebral autoregulation.

**Parameters**	**Stroke ipsilateral** **(*n* = 40)**	**Stroke contralateral** **(*n* = 40)**	**Healthy controls** **(*n* = 18)**
ECP-induced hemodynamics changes			
Mean CBFV at baseline (cm/s)	57.5 ± 22.2	56.3 ± 21.5	57.5 ± 11.9
Mean CBFV during ECP (cm/s)	59.9 ± 22.7	59.4 ± 23.5	56.9 ± 11.3
*P* (baseline vs ECP)	<0.001	<0.001	0.297
CAI (%)	4.4± 5.6^*^	5.3 ± 5.1^*^	−0.7 ± 3.6
Dynamic cerebral autoregulation			
Phase difference (degree)	32.7 ± 39.1^*^	28.6 ± 369^*^	52.6 ± 13.9
Gain (cm/s/mmHg)	1.2 ± 0.9	1.0 ± 0.6	0.9 ± 0.5
Correlation *P* value (CAI vs. phase)	0.326	0.791	0.050
Correlation P value (CAI vs. gain)	0.821	0.389	0.840

*ECP, external counterpulsation; CBFV, cerebral blood flow velocity; CAI, cerebral augmentation index*.

* *P < 0.05 in comparison with healthy controls*.

## Discussion

The present study was a continuation of our previous research work (12, 13, 19, 23). We further correlated the cerebral blood flow augmentation degree induced by ECP as measured by cerebral augmentation index with the impaired dynamic cerebral autoregulation using TFA analysis. We speculate ECP augmented cerebral blood flow of ischemic stroke *via* impaired dynamic cerebral autoregulation, while the degree of ECP-induced cerebral augmentation effects was not related to the magnitude of impaired dynamic cerebral autoregulation.

External counterpulsation increased the mean MCA flow velocities on both ipsilateral and contralateral sides in patients with unilateral ischemic stroke and large artery atherosclerosis. However, it did not change the flow velocities in healthy controls, although mean BP was elevated as well, which was consistent with our previous study (12). Differently, in the same patient cohorts, we investigated their status of dynamic cerebral autoregulation to study the possible hemodynamic mechanisms of augmentation effects following ECP treatment. TFA examines the transfer of the BP oscillations to CBFV as a measure of autoregulation. The autoregulation is supposed to attenuate the influence of ABP on CBFV by preventing a direct transfer of the waveform at a low-frequency range (normally <0.2 Hz). Previous studies of dynamic cerebral autoregulation suggested that autoregulatory parameters in a low-frequency band (0.06–0.12 Hz), used in our study, are more meaningful than in the other frequency bands (24, 25). A coherence threshold of > 0.4 was chosen to define a lower limit of the linearity between arterial BP and CBFV to apply the TFA (15). In the transfer function between BP and CBFV, the gain represents the damping effect of cerebral autoregulation on the magnitude of the BP oscillations. A low gain indicates efficient autoregulation, whereas an increase in gain represents a diminished efficiency of the dynamic process of cerebral autoregulation. PD can be considered a surrogate measure for the time delay of the autoregulatory response. A low PD indicates that CBFV follows the changes in mean BP passively, whereas a higher value of PD suggests that CBFV is actively regulated against the fluctuations of mean BP which represents the better status of autoregulation (26). The PD values on both cerebral sides of patients were significantly lower than that of controls. It indicated that dynamic cerebral autoregulation was bilaterally impaired on the ipsilateral side and contralateral side in the ischemic stroke patients with large artery atherosclerosis, which was comparable with a series of studies on the pattern of dynamic cerebral autoregulation in acute ischemic stroke patients with different subtypes (23, 27).

However, the interesting finding was that the cerebral augmentation index did not correlate with PD and gain on either the ipsilateral or contralateral side of patients with stroke or in controls. It meant the degree of ECP-induced cerebral augmentation effects was not related to the magnitude of impaired dynamic cerebral autoregulation. To the best of our knowledge, two approaches have been introduced to quantify cerebral autoregulation: the static cerebral autoregulation, which reflects the steady-state outcome of cerebral blood flow following a persistent change in BP using thigh cuff inflation, (28, 29) and the dynamic cerebral autoregulation, which investigates the transient relationship between BP and cerebral blood flow based on spontaneous fluctuations of BP (17, 28). To date, little evidence exists concerning the relationship between dynamic and static cerebral autoregulation. Dawson et al. investigated 61 patients with ischemic stroke within 96 h of ictus, and 54 age- and sex-matched controls and found dynamic but not static cerebral autoregulation was globally impaired in acute ischemic stroke (29). One recent study also found no linear correlations between dynamic and static cerebral autoregulation indices in healthy older adults (30). The response of flow velocities following the persistent change in BP induced by ECP may be comparable with the concept of static cerebral autoregulation, which is likely to elucidate a lack of linear correlations between cerebral augmentation effects of ECP and dynamic cerebral autoregulation, quantified by gain and PD in the current study.

The hidden mechanisms are largely unknown. Experimental studies found that ECP reduces endothelial damage, arrests vascular smooth muscle cell proliferation and migration, decreases the proliferating cell nuclear antigen proliferative index, suppresses extracellular matrix formation, and eventually inhibits intimal hyperplasia and the development of atherosclerosis by increasing the arterial wall shear stress, which in turn activates the endothelial-derived nitric oxide (NO) synthase/NO pathway and probably suppresses extracellular signal-regulated kinase 1/2 overactivation (31). In clinical studies on chronic angina (32, 33), a significant increase in plasma NO levels, which is a vasodilator, a decrease in endothelial endothelin-1 (ET-1) levels, which is a vasoconstrictor, and an increase in plasma vascular endothelial growth factor (VEGF), which plays a key role in angiogenesis, were reported after a course of ECP or after the completion of 35 1 h sessions of ECP. Such release of these biomarkers and augmented BP may help open the collateral channels and, thus, augments the collateral perfusion. Although all evidence of biomarker changes now comes from patients with the ischemic disease, we believe the cerebral augmentation effects induced by ECP in patients with stroke should be derived through the same mechanisms. However, there was uncertainty whether 3 min of a single ECP event instead of a series of ECP treatment sessions may produce these beneficial effects. We need to further test the biomarkers released by short-term ECP intervention in patients with acute ischemic stroke. Various neurohumoral, metabolic, and endothelial mechanisms participate in the stability and adjustment of cerebral blood flow (34). Some biomarkers released such as NO and increased activities of the sympathetic control by neurohumoral activation may affect the dynamic cerebral autoregulation, (35). which contributed to the final cerebral augmentation effect degree of ECP.

The limitations of this study included, first, the sample size was relatively small. We failed to subdivide patients with stroke into those with intracranial disease and with the additional extracranial disease, although it may influence cerebral augmentation effects of ECP. Second, age and gender were not comparable between patients with stroke and controls, although they were not the major reason influencing their distinct hemodynamic responses to ECP (36). Third, we independently quantified dynamic cerebral autoregulation using TFA and gain analysis between BP and CBFV fluctuations without ECP intervention, instead of measuring them during ECP. ECP does not compromise the dynamic cerebral autoregulation in healthy persons and patients with atherosclerotic, as revealed by stable values of PD and gain between BP and CBFV oscillations (37). However, the effects of ECP as a treatment on dynamic cerebral autoregulation remain unclear. There is an ongoing randomized controlled trial to investigate the effects of 35 h of daily 1-h ECP treatment sessions on impaired dynamic cerebral autoregulation in patients with ischemic stroke in our center (registration No. ChiCTR-TRC-07000706). The results from that study will be more convincing on whether ECP as a treatment is beneficial for impaired dynamic cerebral autoregulation or not.

In conclusion, the degree of ECP-induced cerebral augmentation effects as measured by the cerebral augmentation index did not correlate with the magnitude of impaired dynamic cerebral autoregulation.

## Data Availability Statement

The raw data supporting the conclusions of this article will be made available by the authors, without undue reservation.

## Ethics Statement

The studies involving human participants were reviewed and approved by Joint CUHK-NTEC Clinical Research Ethics Committee. The patients/participants provided their written informed consent to participate in this study.

## Author Contributions

LX conceived and designed the project and drafted the manuscript. XC helped acquisition of data and interpretation of data. JL helped guide TFA method. LW helped design the project. TL helped recruit participants. All authors had full access to the data, contributed to the study, approved the final version for publication, and take responsibility for its accuracy and integrity.

## Funding

This study was supported by grants from the National Natural Science Foundation of China (No. 82171291), the National Key R&D Program of China (2016YFC1301605), and the Research Grants Council, Hong Kong (CUHK 14100215).

## Conflict of Interest

The authors declare that the research was conducted in the absence of any commercial or financial relationships that could be construed as a potential conflict of interest.

## Publisher's Note

All claims expressed in this article are solely those of the authors and do not necessarily represent those of their affiliated organizations, or those of the publisher, the editors and the reviewers. Any product that may be evaluated in this article, or claim that may be made by its manufacturer, is not guaranteed or endorsed by the publisher.
